# Phenolic Polyketides from the Co-Cultivation of Marine-Derived *Penicillium* sp. WC-29-5 and *Streptomyces* *fradiae* 007

**DOI:** 10.3390/md12042079

**Published:** 2014-04-04

**Authors:** Yi Wang, Liping Wang, Yibin Zhuang, Fandong Kong, Cuixian Zhang, Weiming Zhu

**Affiliations:** 1 Key Laboratory of Marine Drugs, Ministry of Education of China, School of Medicine and Pharmacy, Ocean University of China, Qingdao 266003, China; E-Mails: wangyi0213@hotmail.com (Y.W.); lipingw2006@163.com (L.W.); zhuang_yb@tib.cas.cn (Y.Z.); kongfandong501@126.com (F.K.); 2 School of Chinese Material Medica, Guangzhou University of Chinese Medicine, Guangzhou 510006, China; E-Mail: zhangcuixian@yahoo.com.cn

**Keywords:** *Penicillium* sp*.*, *Streptomyces fradiae*, co-cultivation, secondary metabolites, cytotoxicity

## Abstract

*Penicillium* sp. WC-29-5 was co-cultured with *Streptomyces* *fradiae* 007 to produce five natural products (**1**–**3**, **4a** and **4b**) that were isolated and characterized by spectroscopic analysis. Interestingly, these compounds were found to be different from those produced in discrete fungal and bacterial controls. Among these compounds, the absolute configurations of compounds **4a** and **4b** were determined for the first time by X-ray single crystal diffraction experiments and electronic circular dichroism (ECD) calculations. An evaluation of the cytotoxic activities of these compounds revealed that **4b** was moderately cytotoxic towards HL-60 and H1975 tumor cells with IC_50_ values of 3.73 and 5.73 µM, respectively, whereas compound **4a** was only moderately cytotoxic towards H1975 cells with an IC_50_ value of 3.97 µM.

## 1. Introduction

**Figure 1:**
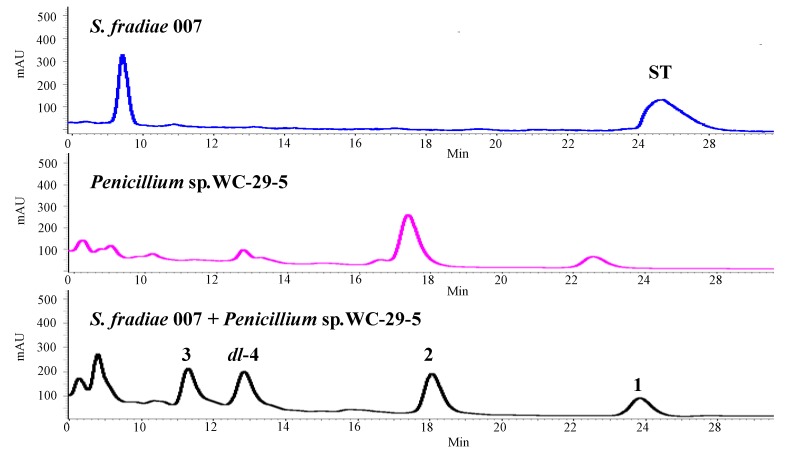
HPLC profiles of secondary metabolites from *S*. *fradiae* 007; *Penicillium* sp. WC-29-5 and co-cultivation of two strains from up to down (HPLC eluent: 0–30 min, 50% MeOH; flow rate: 1 mL/min; detection wavelength: 202 nm).

Natural products have proven to be an incredible resource for drug discovery [1] and the microorganisms responsible for the production of natural products can be considered in some respects as a type of renewable and eco-friendly resource. Many of the microbial gene clusters involved in the synthesis of natural products, however, are silent under traditional lab conditions [2,3,4], and significant research efforts have consequently been devoted to developing new strategies and approaches for activating silent gene clusters to increase the diversity and availability of microbial natural products. It is generally accepted that microbial natural products are used by microbes to communicate with each other, to protect their hosts, or to inhibit the growth of other competitive species in their natural environment [3]. From the perspective of cooperative interactions, the co-cultivation or mixed fermentation of two or more microbes represents a promising approach for simulating physiological conditions and activating silent gene clusters [3,5,6,7]. As part of our ongoing research towards identifying new bioactive natural products from marine-derived microorganisms [8,9,10], we recently became interested in evaluating the mixed fermentation of two microbes and found that the co-cultivation of *Penicillium* sp. WC-29-5 and *Streptomyces* *fradiae* 007 led to the production different metabolites to those produced by each species in isolation (Figure 1). *Streptomyces* *fradiae* is well known for its production of several antibiotics, including neomycin and fosfomycin [11,12]. We previously reported that *S.* *fradiae* 007 could produce staurosporine [13], and the three indolocarbazoles, fradcarbazoles A–C [14]. Herein, we report the isolation, structural elucidation and evaluation of the cytotoxicity of the metabolites produced by the co-cultivation of *Penicillium* sp. WC-29-5 and *S.* *fradiae* 007.

## 2. Results and Discussion

**Figure 2:**
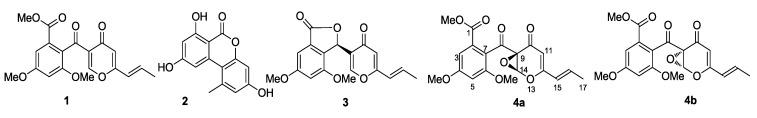
Compounds from the co-cultivation of *Penicillium* sp. WC-29-5 and *S*. *fradiae* 007.

*Penicillium* sp. WC-29-5 and *S*. *fradiae* 007 were co-cultivated in the same medium in the current study to induce the production of different bioactive compounds as a consequence of the competitive nature of the environment. The products formed during the co-cultivation process were investigated at different mix-times. *S.* *fradiae* 007 and *Penicillium* sp. WC-29-5 were cultivated in isolation at 28 °C on a 160 rpm shaker. Seeds of the *S.* *fradiae* 007 (10% inoculum) of different ages (*i.e.*, 0 to 5 day old) were mixed with the same inoculum size of *Penicillium* sp. WC-29-5, and the resulting co-cultures were incubated under the same conditions for 7 days. The results revealed that the growing states and secondary metabolites of the co-cultivated species were the same as those observed for the culturing of *Penicillium* sp. WC-29-5 in isolation when the two strains were seeding at the same time (0 day). In contrast, the growing states and the secondary metabolites observed in the co-cultivation experiments were more like those observed for the culturing of *S.* *fradiae* 007 in isolation when WC-29-5 was added into the 3-day, 4-day and 5-day old cultures of 007, respectively. Significant variations were observed in the secondary metabolites following the seeding of WC-29-5 into a 2-day old culture of 007 (Figure 1). Based on these results, the co-fermentation process was performed by mixing *Penicillium* sp. WC-29-5 with a 2-day old culture of *S.* *fradiae* 007. The resulting mixture was then incubated for 7 days on a 160 rpm shaker at 28 °C. The whole co-fermentation broth (15 L) was extracted thoroughly with ethyl acetate (EtOAc) and the EtOAc extracts were isolated and purified by chromatography, including HPLC, to yield five compounds. By means of spectroscopic analysis, X-ray single crystal diffraction, and theoretical calculations of the electronic circular dichroism (ECD), we have successfully identified these compounds as deoxyfunicone (**1**) [15], alternariol (**2**) [16], vermistatin (**3**) [17], (9*R*,14*S*)-epoxy-11-deoxyfunicone (**4a**), and (9*S*,14*R*)-epoxy-11-deoxyfunicone (**4b**) (Figure 2).

**Figure 3:**
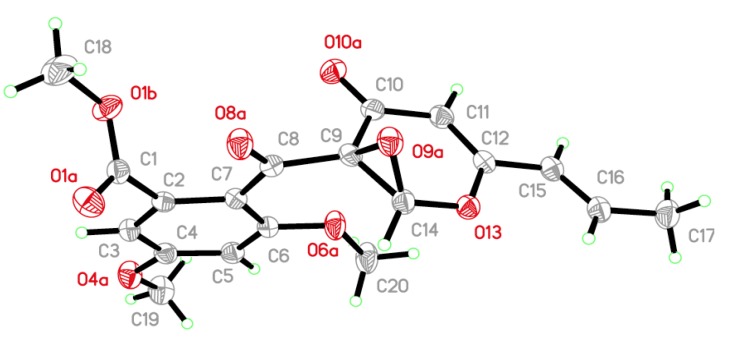
The X-ray crystal structure of the racemic *dl-***4**.

**Figure 4:**
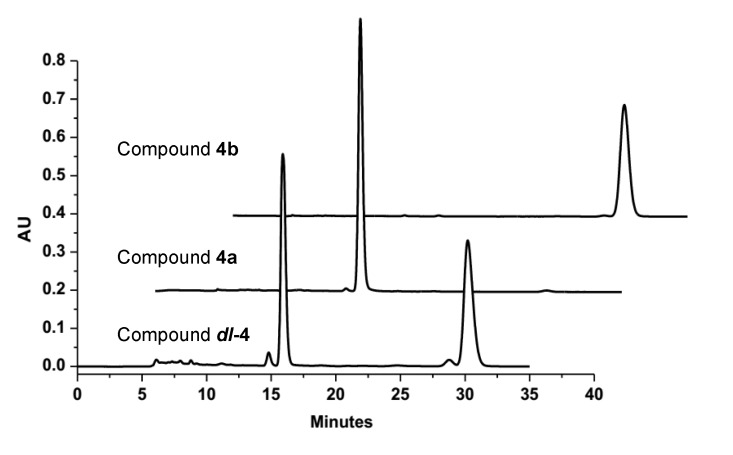
The chiral HPLC analysis and resolution of the racemic *dl-***4** by a Chiralpak IC column (*n*-C_6_H_14_-EtOH 20:80).

**Figure 5:**
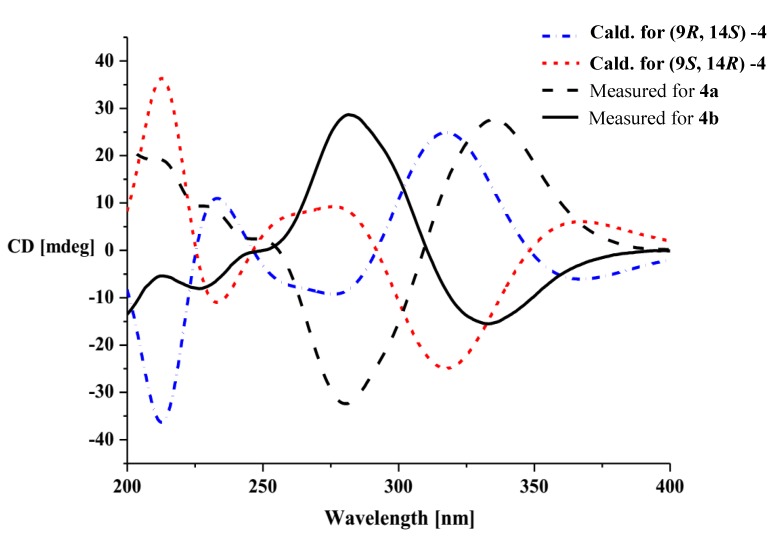
The measured and calculated electronic circular dichroism (ECD) curves of compounds **4a** and **4b**.

**Figure 6:**
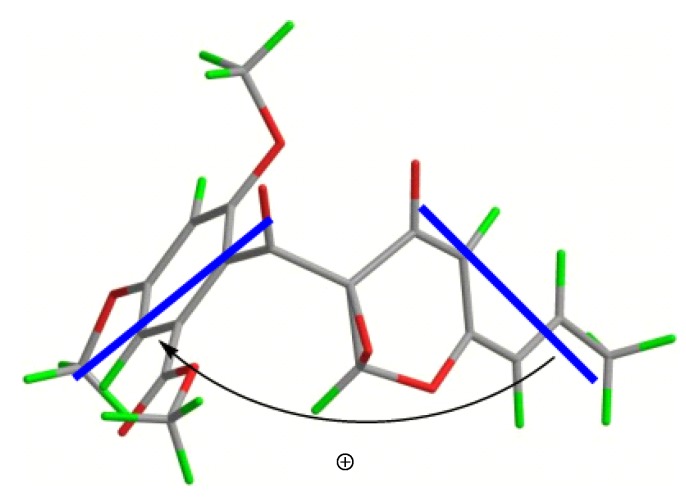
Stereo view of **4a** (bold lines denote the electric transition dipole of the chromophores).

Compounds **4a** and **4b** were initially obtained as a mixture, and analysis by HRESIMS revealed a peak with an *m*/*z* value of 397.0902 [M + Na]^+^, which corresponded to a molecular formula of C_19_H_18_O_8_. Consideration of the literature revealed that the NMR data for this mixture were similar to those reported for 9,14-epoxy-11-deoxyfunicone (Supplementary Information, Table S1, Figures S1–S7), and indicated that compounds **4a** and **4b** were based on the same planar structure [17]. The results of analysis by X-ray single crystal diffraction (Figure 3) also supported this suggestion. The mixture of compounds **4a** and **4b** gave a large Rw (absolute configuration parameter) value and a zero value for its specific rotation, which indicated that these compounds were a racemic mixture. Subsequent analysis by chiral chromatography revealed the presence of two peaks, which were separated to give optically pure **4a** and **4b** (Figure 4). Compounds **4a** and **4b** showed equal and opposite specific rotations (*i.e.*, [α]^23^_D_ +15.8 and −15.4, respectively), which indicated that they were a pair of enantiomers. The CD curves of compounds **4a** and **4b** were measured to determine their absolute configurations, and their ECD curves were also calculated using the time-dependent density functional theory (TD-DFT) method at the B3LYP/6-31G(d) level [18]. Preliminary conformational distribution searches were performed using version 7.5 of the HyperChem software, and the corresponding minimum geometries were fully optimized using DFT at the B3LYP/6-31G(d) level using the Gaussian 03 program package. The results showed that the measured CD curves for **4a** and **4b** were in good agreement with the calculated ECD curves for (9*R*,14*S*)-**4** and (9*S*,14*R*)-**4**, respectively (Figure 5). Furthermore, the CD Cotton effects observed for **4a** and **4b** were attributed to exciton coupling between the transition dipoles of the benzoyl and hexa-2,4-dien-1-one moieties (chromophores). The CD Cotton effect at a long-wave length could be used to predict the absolute configuration according to the Harada-Nakanishi non-empirical rule [19,20]. The positive long-wave Cotton effect of **4a** at λ_max_ 336 nm indicated that the front chromophore existed in a clockwise arrangement relative to the rear chromophore (Figure 6), and implied that compound **4a** was in the (9*R*,14*S*)-configuration. The bisignate CD Cotton effect of **4b** indicated that this compound existed in the opposite configuration to that of **4b**. Thus, compounds **4a** and **4b** were ambiguously determined to be (9*R*,14*S*)- and (9*S*,14*R*)-epoxy-11-deoxyfunicone, respectively.

Stereo-configuration unresolved 9,14-epoxy-11-deoxyfunicone has previously been isolated from the culture of *Talaromyces flavus* IFM52668 and has been reported to be active against *Aspergillus niger* with a 10-mm inhibition zone at 200 µg/disc [17]. To the best of our knowledge, however, there have been no reports in the literature pertaining to the cytotoxicity of this compound towards tumor cell lines. In the current study, we have evaluated the cytotoxicities of optically pure compounds **3**, **4a** and **4b** against HL-60 and H1975 cells using the MTT method. The result of the MTT assay showed that the *d*-isomer **4a** was only moderately active against H1975 tumor cells with an IC_50_ value of 3.97 µM, whereas the *l*-isomer **4b** was moderately active against both two tumor cell lines with IC_50_ values of 3.73 and 5.73 μM, respectively. Compound **3** did not show any cytotoxic activity towards the two tumor cell lines (IC_50_ > 10 μM) (Table 1).

**Table 1:** Cytotoxicities of compounds **3**, **4a** and **4b** against HL-60 and H1975 cells.

Cells	IC_50_ (µM)		
	3	4a	4b
HL-60	>10	>10	3.73
H1975	>10	3.97	5.73

*S.* *fradiae* 007 and *Penicillium* sp. WC-29-5 were incubated separately under the same conditions to those used for the co-cultivation. The co-cultivation process afforded different secondary metabolites to those obtained from the individual fermentation processes (Figure 1). ST, which is the major product of obtained from the cultivation of *S.* *fradiae* 007, was not found in the co-cultivation experiment. Furthermore, compounds **1**, **2**, **4a** and **4b** were only produced in the co-cultivation of *S*. *fradiae* 007 with *Penicillium* sp. WC-29-5. These results suggested that the co-cultivation process was activating silent gene clusters to produce different secondary metabolites.

## 3. Experimental Section

### 3.1. General Experimental Procedures

Specific rotations were obtained on a JASCO P-1020 digital polarimeter. UV spectra were recorded on a Beckman DU 640 spectrophotometer. IR spectra were obtained on a Nicolet NEXUS 470 spectrophotometer as KBr disks. ^1^H and ^13^C NMR analyses, including DEPT spectra and 2D NMR experiments, were recorded on a JEOL JNMECP 600 spectrometer using TMS as internal reference standard. The chemical shifts for the NMR spectra were recorded as *δ* values in ppm. HRESIMS was conducted on a Q-TOF Ultima Global GAA076 LC mass spectrometer (Waters Asia, Ltd., Singapore). CD spectra were measured on a JASCO J-815 spectropolarimeter (Jasco Corporation, Tokyo, Japan). Semipreparative HPLC (Waters Corporation, Milford, MA, USA) was performed using an ODS column (YMC-pack ODS-A, 10 × 250 mm, 5 μm) with a flow rate of 4 mL/min. HPLC was performed using an ODS column (YMC-pack C18, 4.6 × 250 mm, 5 μm) with a flow rate of 2 mL/min. TLC and column chromatography (CC) were performed on plates precoated with silica gel GF254 (10–40 μm) and over silica gel (200–300 mesh, Qingdao Marine Chemical Factory, Qingdao, China) and Sephadex LH-20 (Amersham Biosciences, Uppsala, Sweden), respectively.

### 3.2. Microbial Materials

The fungus *Penicillium* sp.WC-29-5 was isolated from the mangrove soil around the roots of *Aegiceras corniculatum*, Wenchang, Hainan Province of China. It was identified according to its morphological characteristics and 18S rRNA gene sequences (Genbank access No. KJ138167). *Streptomyces* *fradiae* 007 were isolated and identified from a sediment sample collected in Jiaozhou Bay, Shandong Province of China [13,14]. The voucher specimens were deposited in our laboratory at −80 °C.

### 3.3. Fermentation and Extraction

*S.* *fradiae* 007 and *Penicillium* sp. WC-29-5 were separately cultured on an orbital shaker (160 rpm) at 28 °C for 2 days in 500-mL conical flasks containing the liquid medium (100 mL/flask, pH 6.5), which was composed of glucose (10 g/L), maltose (20 g/L), mannitol (20 g/L), monosodium glutamate (10 g/L), yeast extract (3 g/L), KH_2_PO_4_ (0.5 g/L), MgSO_4_•7H_2_O (0.3 g/L) and sea water. *Penicillium* sp. WC-29-5 was then seeded into the *S.* *fradiae* 007 cultures of in 500-mL conical flasks (100 mL/flask, pH 6.5) at 5% of the final concentration for each strain, and the resulting mixtures were co-cultured for 7 days under the same conditions.

The totally cultivated medium (15 L) was harvested and filtered through cheesecloth to separate the broth from the mycelia. The broth was concentrated under reduced pressure to about 5 L and then extracted three times with EtOAc to give an EtOAc solution, whereas the mycelia was extracted three times with acetone. The acetone solution was concentrated under reduced pressure to afford an aqueous solution, which was extracted three times with EtOAc to give a second EtOAc solution. Both EtOAc solutions were combined and concentrated under reduced pressure to give an EtOAc extract (20.0 g). *S.* *fradiae* 007 and *Penicillium* sp. WC-29-5 were also incubated separately under the same conditions as controls. The chemical diversities of the secondary metabolites obtained from the different EtOAc extracts were investigated by HPLC.

### 3.4. Purification and Identification

The EtOAc extract (20.0 g) was separated into nine fractions (fractions 1–9) by CC over silica gel using a stepwise gradient eluting with mixtures of petroleum ether and CHCl_3_ (0%–100%, v/v) and CHCl_3_ and MeOH (0%–100%, v/v). Fraction 6 was separated into seven fractions (fractions 6-1 to 6-7) by CC over silica gel eluting with a step gradient of petroleum ether and acetone (0%–100%, v/v). Fraction 6-6 was separated into five fractions on a Sephadex LH-20 column using a mixture of MeOH and CHCl_3_ (1:1, v/v) as the eluent. Fraction 6-6-2 was further purified by semipreparative HPLC with 70% MeOH to give **1** (8.1 mg, *t*_R_ 12.8 min). Fraction 6-7 was also purified over a Sephadex LH-20 column eluting with a mixture of MeOH and CHCl_3_ (1:1, v/v) and 100% MeOH, before being further purified by semipreparative HPLC with 50% MeOH to give compounds **3** (11.4 mg, *t*_R_ 11.2 min) and *dl-***4** (3 mg, *t*_R_ 12.9 min). The racemic *dl-***4** was purified over a Chiralpak IC column eluting with n-hexane and EtOH (20:80, v/v) to give the pure compounds **4a** (0.8 mg, *t*_R_ 15.9 min) and **4b** (0.8 mg, *t*_R_ 30.2 min). Fraction 7 was purified by CC over silica gel using a step gradient elution with petroleum ether and acetone (0%–100%, v/v) to give five fractions (fraction 7-1 to 7-5). Fraction 7-5 was also purified over a Sephadex LH-20 column eluting with MeOH and CHCl_3_ (1:1, v/v), before being further purified by semipreparative HPLC with 60% MeOH to give **2** (4.0 mg, *t*_R_ 10.2 min).

(*dl*)-Epoxy-11-deoxyfunicone (*dl-***4**): colorless needles, mp 166 °C (hexane-EtOAc), IR (KBr) ν_max_ 1731, 1670, 1649, 1646, 1363, 1216, 1154, 1058 and 905 cm^−1^; HRESI-MS *m*/*z* 397.0902 [M + Na]^+^. ^1^H NMR (600 MHz) and ^13^C NMR (150 MHz) see Table S1.

(9*R*,14*S*)-Epoxy-11-deoxyfunicone (**4a**): [α]^23^_D_ +15.8 (*c* 0.04, MeOH); UV (MeOH) λ_max_ (log ε) 208 (4.18), 238 (3.90) and 286 (4.03) nm; CD (*c* 0.04, MeOH) λ_max_ (Δε) 212 (+3.1), 280 (−7.4) and 336 (+6.1) nm.

(9*S*,14*R*)-Epoxy-11-deoxyfunicone (**4b**): [α]^23^_D_ −15.4 (*c* 0.04, MeOH); UV (MeOH) λ_max_ (log ε) 208 (4.18), 238 (3.90) and 286 (4.03) nm; CD (*c* 0.04, MeOH) λ_max_ (Δε) 212 (−2.9), 280 (+7.8) and 336 (−5.8) nm.

X-ray crystal data for (*dl*)-epoxy-11-deoxyfunicone (*dl-***4**): Racemic *dl-***4** was obtained as a colorless orthorhombic crystal from a mixture of MeOH and CHCl_3_ (1:1, v/v): molecular formula C_19_H_18_O_8_; space group *P*2_1_2_1_2_1_ with *a* = 7.8524(10) Å, *b* = 15.0361(2) Å, *c* = 15.2591(2) Å, *V* = 1801.63(4) Å^3^, *Z* = 4, *D*_calcd._ = 1.380 mg/m^3^, *μ* = 0.922 mm^−1^, and *F*(000) = 784; crystal size: 0.60 × 0.40 × 0.40 mm^3^; Absolute structure parameter: 0.25(13). *T* = 150(2) K. A total of 16,620 unique reflections (2*θ* < 50°) were collected on a CrysAlis PRO CCD area detector diffractometer with graphite-monochromated Cu Kα radiation (λ = 1.54184 Å). The structure was solved by direct methods (SHELXS-97) and expanded using Fourier techniques (SHELXL-97). The final cycle of full-matrix least-squares refinement was based on 3401 unique reflections (2*θ* < 50°) and 248 variable parameters and converged with unweighted and weighted agreement factors of R1 = 0.0288 and R2 = 0.0736 for I > 2*σ*(*I*) data. Crystallographic data for *dl-***4** have been deposited with the Cambridge Crystallographic Data Centre (No. CCDC 856489). Copies of the data can be obtained, free of charge, on application to the CCDC, 12 Union Road, Cambridge CB2 1EZ, UK.

### 3.5. Bioassay

The cytotoxic effects of the compounds on HL-60 and H975 tumor cells were evaluated using the MTT assay method [21]. Briefly, the cell lines were grown in RPMI-1640 medium supplemented with 10% FBS under a humidified atmosphere of 5% CO_2_ and 95% air at 37 °C. Cell suspensions of 200 μL in size at a density of 5 × 10^4^ cell mL^−1^ were plated in 96-well microtiter plates and incubated for 24 h at 37 °C. Portions of the test solutions (2 μL in MeOH) were then added to each well and the resulting mixtures were incubated for 72 h. MTT solution (20 μL, 5 mg/mL in IPMI-1640 medium) was then added to each well, and the resulting mixtures were incubated for 4 h. The old media containing the MTT (150 μL) were then gently replaced with DMSO and pipetted to allow for the dissolution of the formazan crystals. The absorbance values were then determined on a SpectraMax Plus plate reader at 540 nm. The IC_50_ values were calculated as the concentration of compound required to reduce cell viability by 50%.

## 4. Conclusions

In contrast to the discrete fungal and bacterial controls, the co-fermentation of *Penicillium* sp. WC-29-5 with *Streptomyces* *fradiae* 007 led to the production of four completely aromatic polyketides, including deoxyfunicone (**1**), 1,3,8-trihydroxy-6-methylxanthen-9-one (**2**), (9*R*,14*S*)-epoxy-11-deoxy funicone (**4a**) and (9*S*,14*R*)-epoxy-11-deoxyfunicone (**4b**). Furthermore, the absolute configurations of compounds **4a** and **4b** as well as their cytotoxicities against HL-60 and H1975 cells have been reported in this study for the first time. The results of the current study demonstrate that the co-culturing of two microbes can induce the synthesis of secondary metabolites that would otherwise not be formed during the cultivation of the same species in isolation. Furthermore, the microorganisms used in the current study survived in a competitive environment and produced bioactive secondary metabolites.

## Supplementary Files

Supplementary File 1 Supplementary Information (PDF, 552 KB)
