# Supplementary material for: Phenolic Polyketides from the Co-Cultivation of Marine-Derived Penicillium sp. WC-29-5 and Streptomyces fradiae 007
Source: Mar Drugs. 2014 Apr 4;12(4):2079–88. doi: 10.3390/md12042079 (PMC4012438; doi:10.3390/md12042079)
Supplement: Supplementary File 1 — Supplementary Information (PDF, 552 KB) [file marinedrugs-12-02079-s001.pdf]

Supplementary Information

S1. Theory and Calculation Details

The calculations were performed by using the density functional theory (DFT) as carried out in the Gaussian 03 [1]. The preliminary conformational distributions search was performed by HyperChem 7.5 software. All ground-state geometries were optimized at the B3LYP/6-31G(d) level. Solvent effects of methanol solution were evaluated at the same DFT level by using the SCRF/PCM method [2–4]. TDDFT [5–8] at B3LYP/6-31G(d) was employed to calculate the electronic excitation energies and rotational strengths in methanol.

S2. The 18S rRNA Gene Sequences Data of *Penicillium* sp. WC-29-5

AGCACTTTATACTGTGAAACTGCGAATGGCTCATTAAATCAGTTATCGTTTATTTGATAGTACCTTACTACA
TGGATACCTGTGGTAATTCTAGAGCTAATACATGCTACAAACCCCGACTTCAGGAAGGGGTGTATTTATTA
GATAAAAAACCAACGCCCTTCGGGGCTCCTTGGTGAATCATAATAACTTAACGAATCGCATGGCCTTGCG
CCGGCGATGGTTCATTCAAATTTCTGCCCTATCAACTTTCGATGGTAGGATAGTGGCCTACCATGGTGGCA
ACGGGTAACGGGGAATTAGGGTTCGATTCCGGAGAGGGAGCCTGAGAAACGGCTACCACATCCAAGGA
AGGCAGCAGGCGCGCAAATTACCCAATCCCGATACGGGGAGGTAGTGACAATAAATACTGATACGGGGC
TCTTTCGGGTCTCGTAATTGGAATGAGAACAATTTAAATCCCTTAACGAGGAACAATTGGAGGGCAAGTC
TGGTGCCAGCAGCCGCGGTAATTCCAGCTCCAATAGCGTATATTAAAGTTGTTGCAGTTAAAAAGCTCGT
AGTTGAACCTTGGGCCTGGCTGGCCGGTCCGCCTCACCGCGAGTACTGGTCCGGCTGGGCCTTTCCTTCT
GGGGAACCTCATGGCCTTCACTGGCTGTGGGGGGAACCAGGACTTTTACTGTGAAAAAATTAGAGTGTT
CAAAGCAGGCCTTTGCTCGAATACATTAGCATGGAATAATAGAATAGGACGTGCGGTTCTATTTTGTTGGT
TTCTAGGACCGCCGTAATGATTAATAGGGATAGTCGGGGGCGTCAGTATTCAGCTGTCAGAGGTGAAATT
CTTGGATTTGCTGAAGACTAACTACTGCGAAAGCATTCGCCAAGGATGTTTTCATTAATCAGGGAACGAA
AGTTAGGGGATCGAAGACGATCAGATACCGTCGTAGTCTTAACCATAAACTATGCCGACTAGGGATCGGA
CGGGATTCTATGATGACCCGTTCGGCACCTTACGAGAAATCAAAGTTTTTGGGTTCTGGGGGGAGTATGG
TCGCAAGGCTGAAACTTAAAGAAATTGACGGAAGGGCACCACAAGGCGTGGAGCCTGCGGCTTAATTTG
ACTCAACACGGGGAAACTCACCAGGTCCAGACAAAATAAGGATTGACAGATTGAGAGCTCTTTCTTGAT
CTTTTGGATGGTGGTGCATGGCCGTTCTTAGTTGGTGGAGTGATTTGTCTGCTTAATTGCGATAACGAACG
AGACCTCGGCCCTTAAATAGCCCGGTCCGCATCTGCGGGCCGCTGGCTCTTAGGGGACTATCGCTCAGCC

Deoxyfunicone (**1**): $C_{19}H_{18}O_7$, light yellow needles, mp 130–132 ℃ (*n*-C_6H_{14}/C_6H_6); ESI-MS *m*/*z* 359.1 $[M + H]^+$ ^{1}H NMR (600 MHz, $CDCl_3$) and ^{13}C NMR (150 MHz, $CDCl_3$) see Table S1.

Alternariol (**2**): $C_{14}H_{10}O_5$, light yellow solid, ESI-MS *m*/*z* 257.1 $[M - H]^+$ ^{1}H NMR (600 MHz, DMSO-d_6) and ^{13}C NMR (150 MHz, DMSO-d_6) see Table S1.

Vermistatin (**3**): $C_{18}H_{16}O_6$, light yellow needles, mp 211–212 ℃ (MeOH), $[\alpha]_D^{25}$ −82 °(*c* 0.5, MeOH); ESI-MS *m*/*z* 329.1 $[M + H]^+$ ^{1}H NMR (600 MHz, $CDCl_3$) and ^{13}C NMR (150 MHz, $CDCl_3$) see Table S1.

Figure S1. The Physicochemical Data of the Known Compounds **1**–**3**.

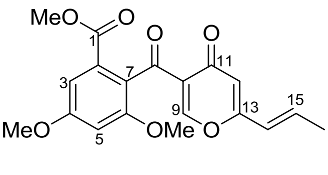

1

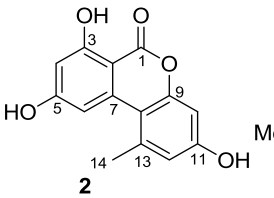

2

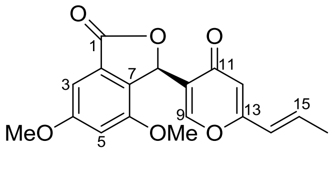

3

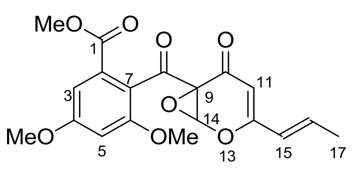

dl-**4**

Table S1. The ^{1}H and ^{13}C NMR (600 and 150 MHz) data for compounds **1**, **3**, and *dl*-**4** in $CDCl_3$ and **2** in DMSO-d_6.

Position	1		2		3		*dl*-4	
	δ_C	δ_H (*J* in Hz)	δ_C	δ_H (*J* in Hz)	δ_C	δ_H (*J* in Hz)	δ_C	δ_H (*J* in Hz)
1	166.6, C_q	-	166.0, C_q	-	170.2, C_q	-	159.2, C_q	-
2	130.6, C_q	-	98.0, C_q	-	129.5, C_q	-	134.2, C_q	-
3	106.0, CH	6.94 (1H, d, 2.3)	165.3, C	-	99.1, CH	6.98 (1H, d, 2.3)	101.5, CH	6.53 (1H, d, 2.3)
4	161.7, C_q	-	101.4, CH	6.36 (1H, d, 2.2)	163.2, C_q	-	163.2, C_q	-
5	103.2, CH	6.84 (1H, d, 2.3)	164.6, C_q	-	105.2, CH	6.68 (1H, d, 2.3)	106.2, CH	6.86 (1H, d, 2.3)
6	158.2, C_q	-	104.9, CH	7.24 (1H, d, 2.2)	155.0, C_q	-	167.8, C_q	-
7	126.6, C_q	-	138.7, C_q	-	123.5, C_q	-	119.7, C_q	-
8	191.0, C_q	-	109.5, C_q	-	73.7, CH	6.15 (1H, s)	185.5, C_q	-
9	161.4, CH	8.60 (1H, s)	153.2, C_q	-	154.0, CH	7.42 (1H, s)	62.8, C_q	-
10	126.0, C_q	-	102.2, CH	6.63 (1H, d, 2.5)	127.8, C_q	-	190.8, C_q	-
11	175.3, C_q	-	159.0, C_q		177.4, C_q	-	104.1, CH	5.47 (1H, s)
12	114.7, CH	6.26 (1H, s)	118.2, CH	6.71 (1H, d, 2.5)	113.0, CH	6.45 (1H, s)	161.4, C_q	-
13	161.2, C_q	-	138.9, C_q		163.2, C_q	-	-	-
14	123.2, CH	6.30 (1H, dq, 15.4, 1.8)	25.8, CH_3	2.70 (3H, s)	123.2, CH	6.06 (1H, dq ,15.6,1.8)	81.6, CH	5.60 (1H, s)
15	136.7, CH	6.68 (1H, dq, 15.4, 6.8)	-	-	136.0, CH	6.60 (1H, dq, 15.6, 6.8)	124.1, CH	5.95 (1H, dq, 15.4,1.4)
16	18.8, CH_3	1.90 (3H, dd, 6.8,1.8)	-	-	18.7, CH_3	1.92 (3H, dd, 6.8, 1.8)	137.9, CH	6.67 (1H, dq, 15.4, 6.8)
17	-	-	-	-	-	-	18.7, CH_3	1.92 (3H, dd, 6.8, 1.4)
1-OCH_3	56.8, CH_3	3.85 (3H, s)	3-OH	11.69 (1H, s)	-	-	56.1, CH_3	3.75 (3H, s)
4-OCH_3	56.3, CH_3	3.70 (3H, s)	5-OH	10.85 (1H, s)	55.9, CH_3	3.78 (3H, s)	55.9, CH_3	3.85 (3H, s)
6-OCH_3	52.9, CH_3	3.70 (3H, s)	11-OH	10.28 (1H, s)	56.1, CH_3	3.87 (3H, s)	53.0, CH_3	3.85 (3H, s)

Figure S2. The ^{1}H NMR spectrum of the racemic *dl*-**4** in $CDCl_3$.

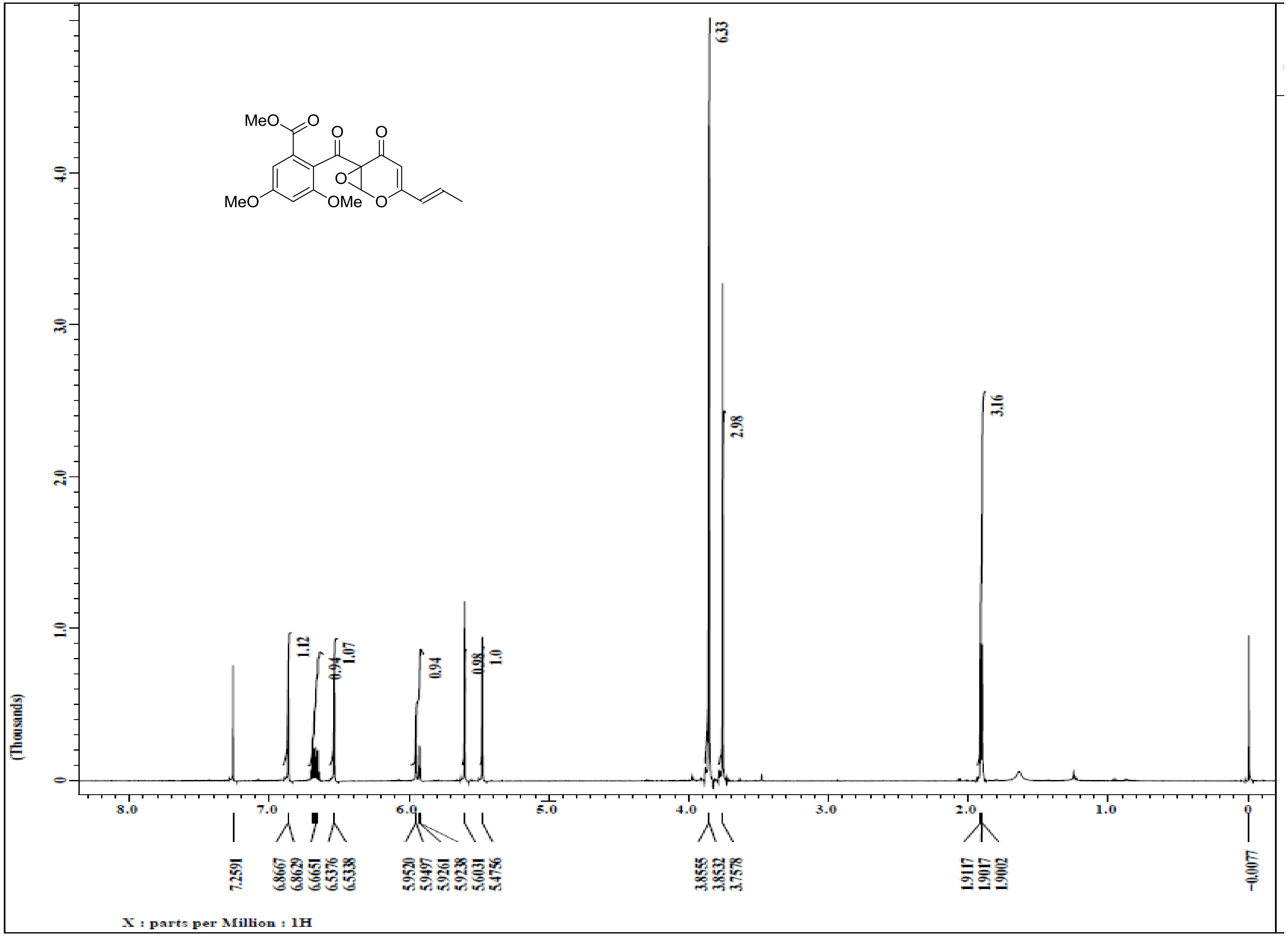

Figure S3. The ^{13}C NMR spectrum of the racemic *dl*-**4** in $CDCl_3$.

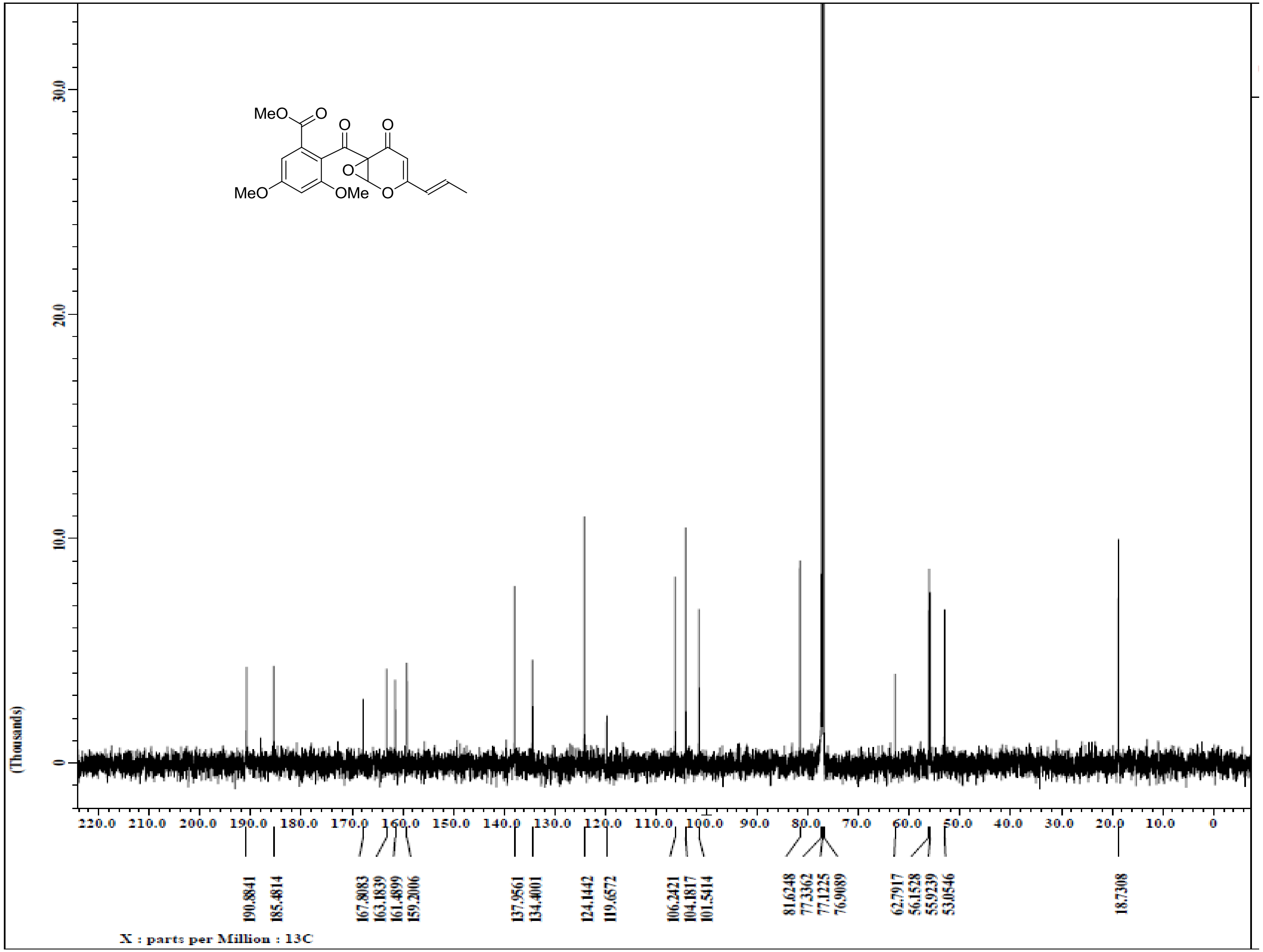

Figure S4. The DEPT spectrum of the racemic *dl*-**4** in $CDCl_3$.

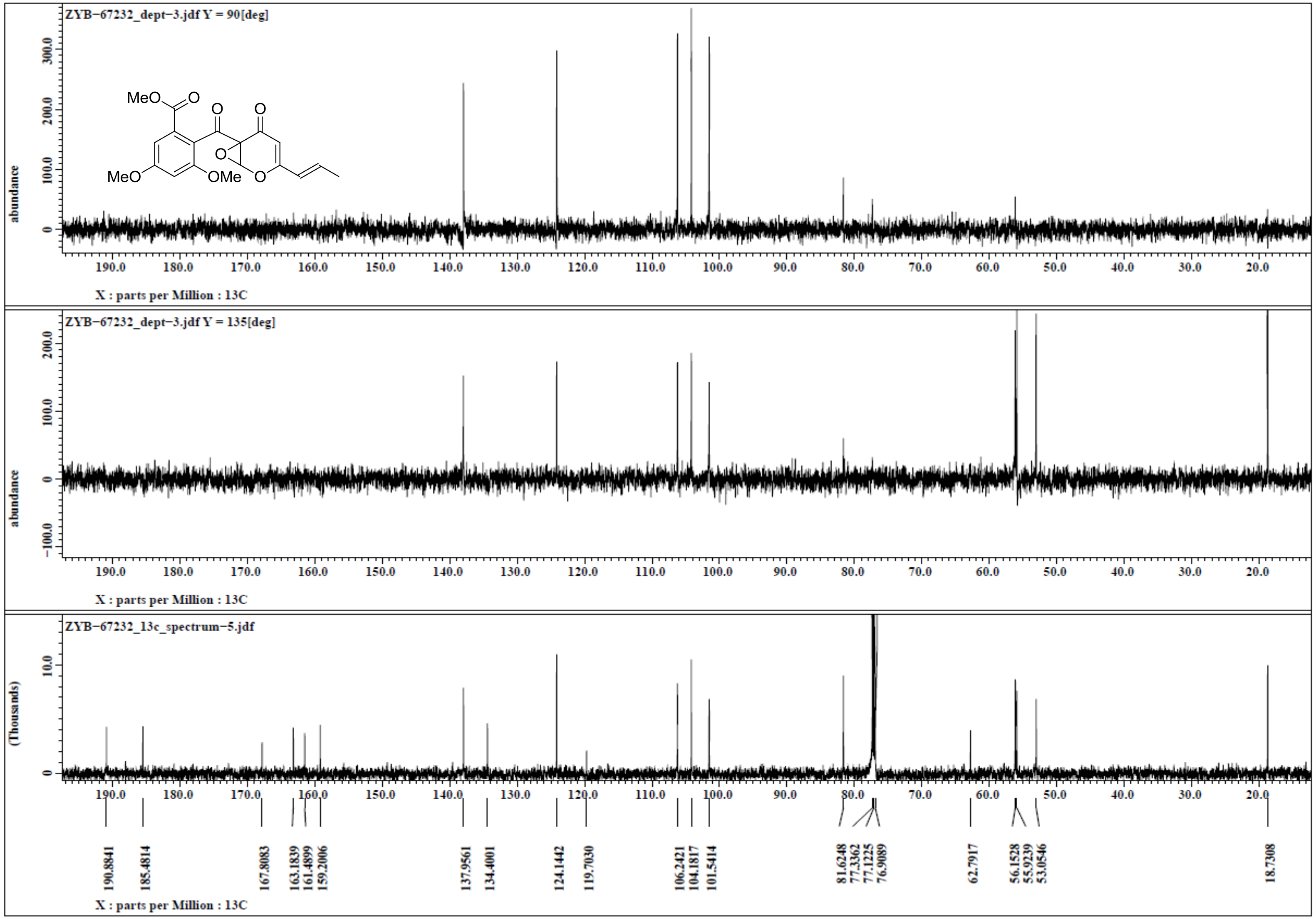

Figure S5. The HSQC spectrum of the racemic *dl*-**4** in $CDCl_3$.

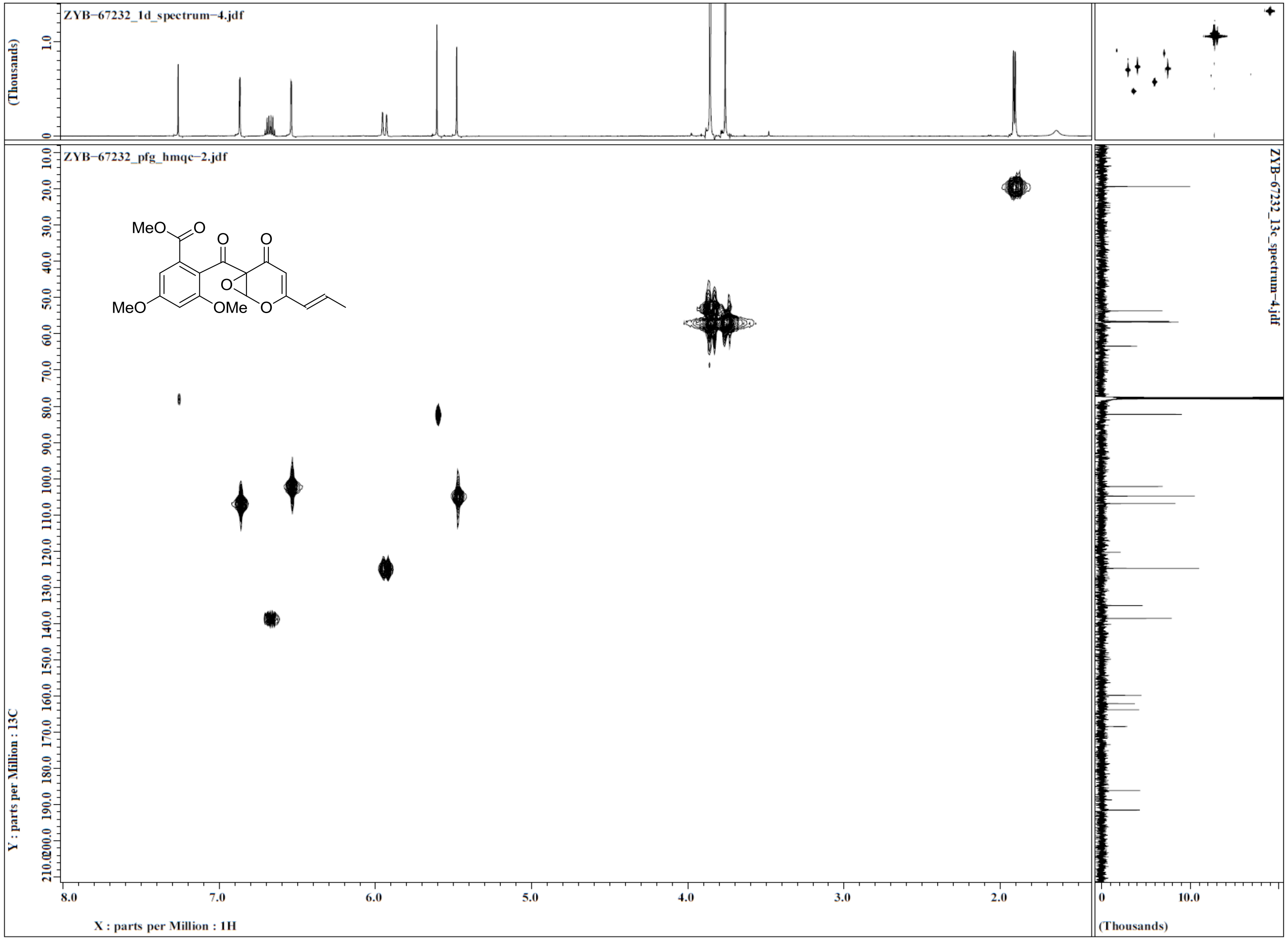

Figure S6. The ^{1}H-^{1}H COSY spectrum of the racemic *dl*-**4** in $CDCl_3$.

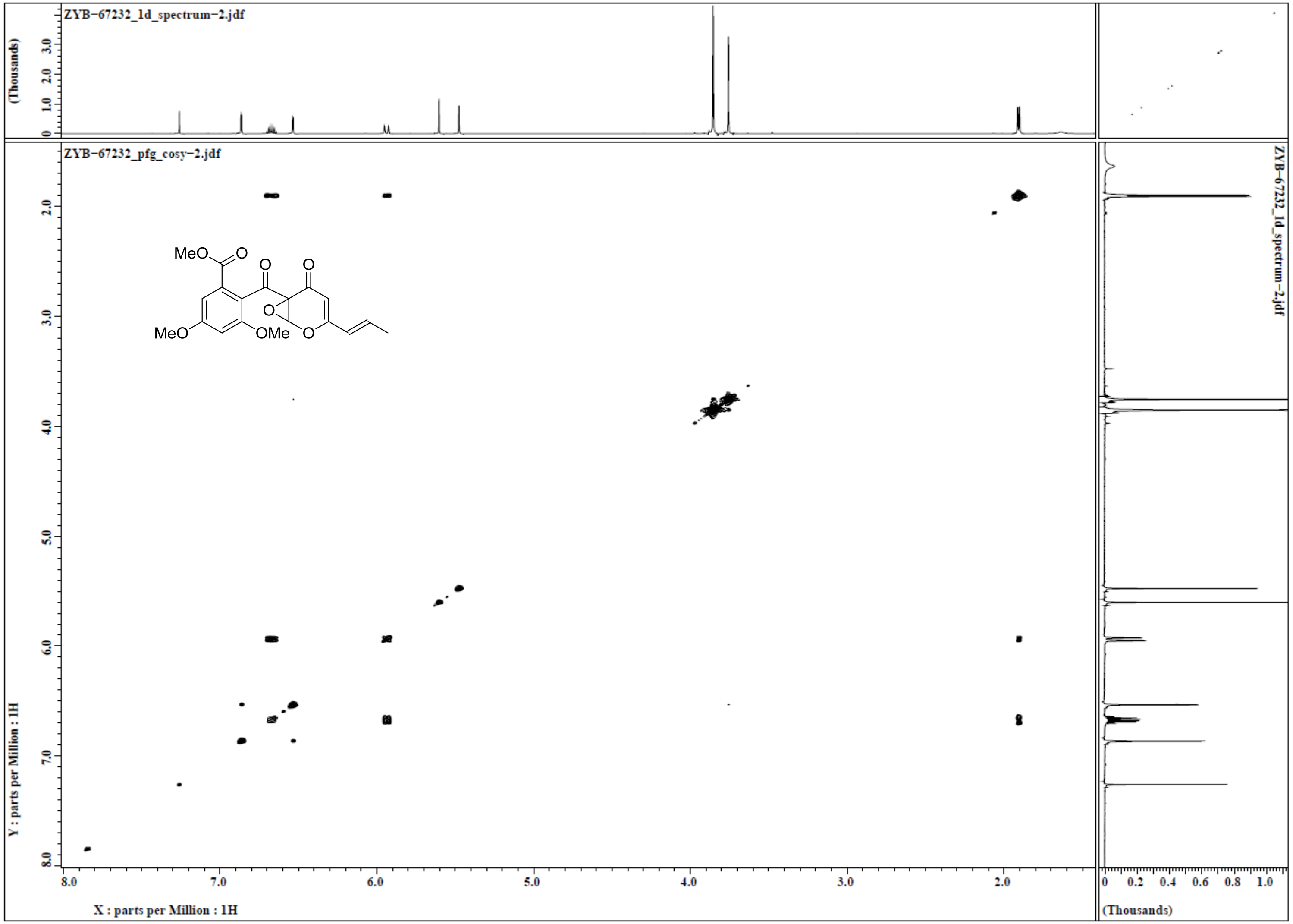

Figure S7. The HMBC spectrum of the racemic *dl*-**4** in $CDCl_3$.

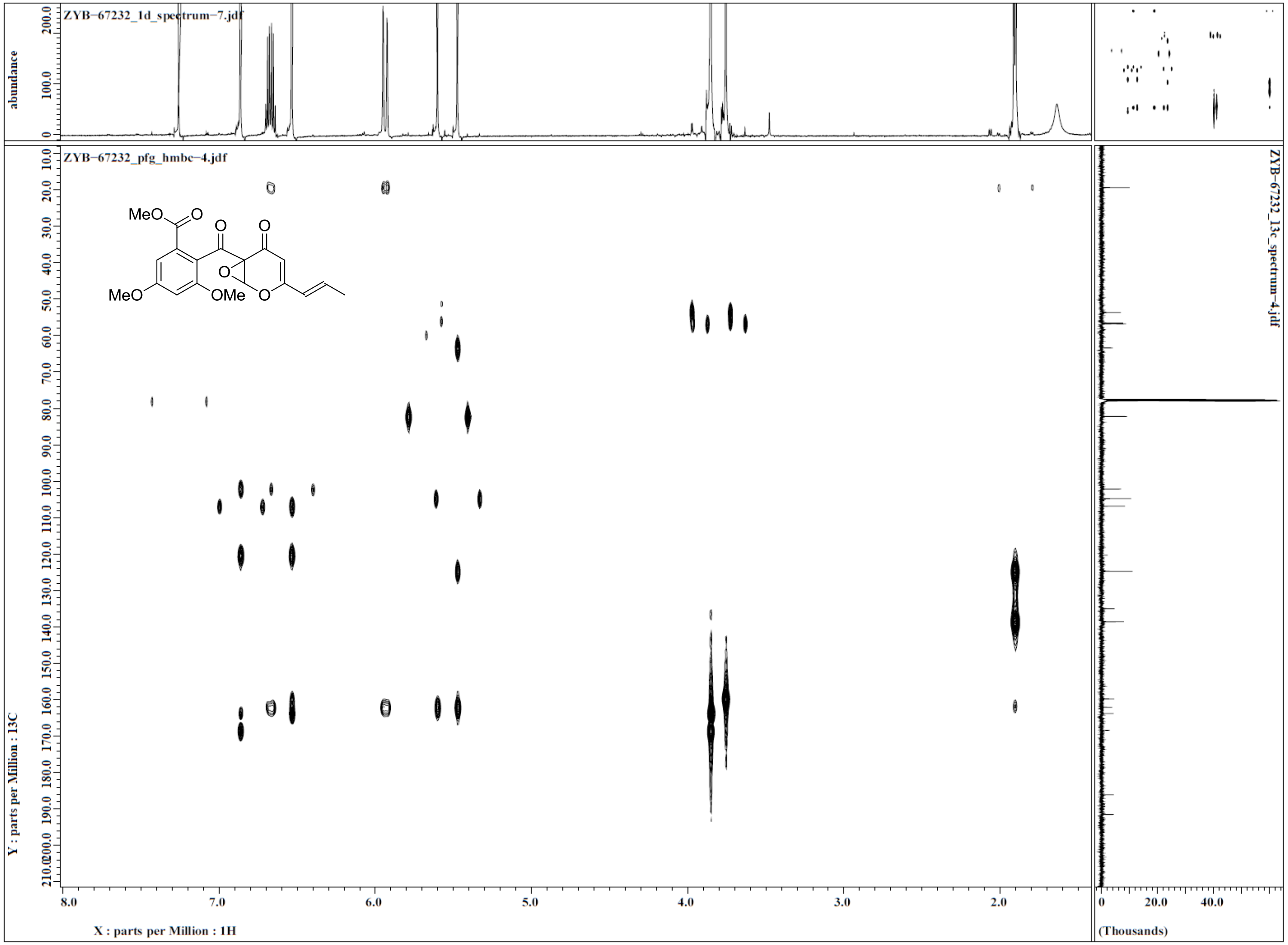

References

1. Frisch, M.J.; Trucks, G.W.; Schlegel, H.B.; Scuseria, G.; ERobb, M.A.; Cheeseman, J.R.; Montgomery, J.A., Jr.; Vreven, T.; Kudin, K.N.; Burant, J.C.; *et al. Gaussian 03, Revision E.01*; Gaussian, Inc.: Wallingford, CT, USA, 2004.
2. Miertus, S.; Tomasi, J. Approximate evaluations of the electrostatic free energy and internal energy changes in solution processes. *Chem. Phys.* **1982**, *65*, 239–245.
3. Tomasi, J.; Persico, M. Molecular interactions in solution: An overview of methods based on continuous distributions of the solvent. *Chem. Rev.* **1994**, *94*, 2027–2094.
4. Cammi, R.; Tomasi, J. Remarks on the use of the apparent surface charges (ASC) methods in solvation problems: Iterative versus matrix-inversion procedures and the renormalization of the apparent charges. *J. Comp. Chem.* **1995**, *16*, 1449–1458.
5. Casida, M.E. *Recent Advances in Density Functional Methods, Part I*; Chong, D.P., Ed.; World Scientific: Singapore, Singapore, 1995; pp. 155–192.
6. Gross, E.K.U.; Dobson, J.F.; Petersilka, M. Density functional theory II. *Top. Curr. Chem.* **1996**, *181*, 81–172.
7. Gross, E.K.U.; Kohn, W. Time-dependent density-functional theory. *Adv. Quant. Chem.* **1990**, *21*, 255–291.
8. Runge, E.; Gross, E.K.U. Density-functional theory for time-dependent systems. *Phys. Rev. Lett.* **1984**, *52*, 997–1000.

© 2014 by the authors; licensee MDPI, Basel, Switzerland. This article is an open access article distributed under the terms and conditions of the Creative Commons Attribution license (http://creativecommons.org/licenses/by/3.0/).
